# A systematic review of geographical differences in management and outcomes for colorectal cancer in Australia

**DOI:** 10.1186/s12885-017-3067-1

**Published:** 2017-02-02

**Authors:** Michael J. Ireland, Sonja March, Fiona Crawford-Williams, Mandy Cassimatis, Joanne F. Aitken, Melissa K. Hyde, Suzanne K. Chambers, Jiandong Sun, Jeff Dunn

**Affiliations:** 1 Institute of Resilient Regions, University of Southern Queensland, Springfield Central, Australia; 2 School of Psychology and Counselling, University of Southern Queensland, Springfield Central, Australia; 3 Non-communicable Disease Control Unit, Melbourne School of Population and Global Health, University of Melbourne, Melbourne, VIC Australia; 4 Cancer Research Centre, Cancer Council Queensland, Fortitude Valley, 4006 QLD Australia; 5 School of Public Health and Social Work, Queensland University of Technology, Brisbane, Australia; 6 Menzies Health Institute Queensland, Griffith University, Brisbane, QLD Australia; 7 Menzies Health Institute Queensland, Griffith University, Southport, QLD Australia; 8 Prostate Cancer Foundation of Australia, St Leonards, NSW Australia; 9 Exercise Medicine Research Institute, Edith Cowan University, Perth, WA Australia; 10 School of Social Science, University of Queensland, Brisbane, Australia; 11 School of Medicine, Griffith University, Brisbane, QLD Australia

**Keywords:** Bowel cancer, Colorectal cancer, Disparity, Regional, Health outcome

## Abstract

**Background:**

Australia and New Zealand have the highest incidence of colorectal cancer (CRC) in the world, presenting considerable health, economic, and societal burden. Over a third of the Australian population live in regional areas and research has shown they experience a range of health disadvantages that result in a higher disease burden and lower life expectancy. The extent to which geographical disparities exist in CRC management and outcomes has not been systematically explored. The present review aims to identify the nature of geographical disparities in CRC survival, clinical management, and psychosocial outcomes.

**Methods:**

The review followed PRISMA guidelines and searches were undertaken using seven databases covering articles between 1 January 1990 and 20 April 2016 in an Australian setting. Inclusion criteria stipulated studies had to be peer-reviewed, in English, reporting data from Australia on CRC patients and relevant to one of fourteen questions examining geographical variations in a) survival outcomes, b) patient and cancer characteristics, c) diagnostic and treatment characteristics and d) psychosocial and quality of life outcomes.

**Results:**

Thirty-eight quantitative, two qualitative, and three mixed-methods studies met review criteria. Twenty-seven studies were of high quality, sixteen studies were of moderate quality, and no studies were found to be low quality. Individuals with CRC living in regional, rural, and remote areas of Australia showed poorer survival and experienced less optimal clinical management. However, this effect is likely moderated by a range of other factors (e.g., SES, age, gender) and did appear to vary linearly with increasing distance from metropolitan centres. No studies examined differences in use of stoma, or support with stomas, by geographic location.

**Conclusions:**

Overall, despite evidence of disparity in CRC survival and clinical management across geographic locations, the evidence was limited and at times inconsistent. Further, access to treatment and services may not be the main driver of disparities, with individual patient characteristics and type of region also playing an important role.

A better understanding of factors driving ongoing and significant geographical disparities in cancer related outcomes is required to inform the development of effective interventions to improve the health and welfare of regional Australians.

## Background

Australia and New Zealand have the highest incidence of colorectal cancer (CRC) in the world, with approximately 1 in 13 Australians likely to develop CRC in their lifetime [1, 2]. CRC has the second-highest incidence of all types of cancers in Australia, after prostate cancer with an estimated 14,958 people diagnosed in 2012, and with incidence rates expected to increase [1]. CRC is responsible for the second-highest burden of disease attributable to cancer in Australia and in 2008–09, accounted for the highest expenditure of any cancer costing the Australian government $427 million in tangible and intangible costs (e.g., screening programs, hospital services, pharmaceuticals, etc., [3]). Thus, the burden of CRC on the Australian health care system is substantial and increasing.

Despite having one of the most urbanised populations in the world (89.2%), over a third (approximately 8 million) of Australians live in non-metropolitan locations classified as regional, rural, or remote [4]. Regional Australians face a range of health disadvantages that result in greater disease burden and lower life expectancy [5, 6]. In light of the high prevalence and disease burden of CRC within Australia, geographic variation in CRC incidence, management, and outcomes is an important question.

CRC can develop without early warning signs. If detected early, CRC is very treatable as polyps can be removed with a minimally invasive day procedure [7]. Therefore, early detection is essential to provide the best treatment outcomes. In response to the proven effectiveness of screening in reducing CRC mortality [8–10], the Australian government introduced the National Bowel Cancer Screening Program (NBCSP) in 2006. The program involves mailing Australians aged >50 years an immunochemical Faecal Occult Blood Test (FOBT) kit. Deaths from CRC in the United States have decreased with the use of screening tools such as colonoscopies and FOBTs [11]. However, in Australia, there remains a relatively low rate of test completion with participation rates appearing to be particularly low in remote and very remote areas [12].

A number of social groups experience disadvantage in cancer care in terms of preventative actions, access to recommended and timely treatment, psychosocial support, and specialist care [12–15]. For example, Australians residing in rural and remote areas may experience disadvantage in cancer care relative to metropolitan residents; while Indigenous Australians may be more likely to experience cancer care disadvantage relative to Caucasian Australians. Clinical outcomes indicate that geographical remoteness and Indigenous status may result in poorer treatment and survival outcomes [16, 17]. Reasons for this disadvantage are many and complex, and while cultural barriers and a lack of access to services undoubtedly play a role, these are unlikely to be the only factors operating to produce disparities. Additional patient, professional, and system factors affect outcomes, although the relationships between these are also likely to be complex. The determination of the role of these factors is required to ensure the best possible outcomes for patients. However, no comprehensive synthesis of the available evidence has been published.

The present review aimed to identify the nature of geographical disparity in CRC survival, screening, treatment, clinical management, and psychosocial outcomes. An additional aim was to uncover broad trends in the focus of published research addressing issues of geographical disparity relating to CRC in the Australian context. We anticipate that by identifying patterns of covarying disparities across the domains reviewed, we may be able to speculate about possible causes and make recommendations for future research to explore these.

### Review questions

Questions to guide this review were developed by a Project Steering Committee that included clinicians, researchers, allied health practitioners, and stakeholder representatives (Cancer Council Queensland). Research questions were based on a preliminary scoping review of CRC outcome research and formulated following the PICO framework [18]. The 14 questions are reported in Table 1 and can be grouped according to four themes 1. Survival outcomes (1 question), 2. Patient and cancer characteristics (2 questions), 3. Diagnostic and treatment characteristics (7 questions) and 4. Psychosocial and quality of life outcomes (4 questions).

**Table 1:** Clinical questions

Survival outcomes	Q1. For individuals diagnosed with colorectal cancer, do those who reside in non-metropolitan areas have poorer survival rates than those living in metropolitan areas in Australia?
Patient and cancer characteristics	Q2. For individuals diagnosed with colorectal cancer, do non-metropolitan populations have different sociodemographic characteristics compared with metropolitan populations in Australia?
	Q3. For individuals diagnosed with colorectal cancer, do those living in non-metropolitan areas have a more advanced stage of cancer at diagnosis compared with people living in metropolitan Australia?
Diagnostic and treatment characteristics	Q4. For individuals who are in the colorectal cancer screening target group, are those residing in non-metropolitan areas less likely to access screening services compared with people residing in metropolitan areas of Australia?
	Q5. For individuals with colorectal cancer, are there differences in the clinical management of those who reside in non-metropolitan areas and people residing in metropolitan areas of Australia?
	Q6. Are individuals with colorectal cancer who live in non-metropolitan areas less likely to receive recommended clinical management compared with those who live in metropolitan areas in Australia?
	Q7. For individuals who have colorectal cancer, are those who live in non-metropolitan areas less likely to complete prescribed treatment than those who live in metropolitan areas in Australia?
	Q8. For individuals with colorectal cancer, are those in non-metropolitan areas more likely to experience delays in referral to, and examination by, colorectal cancer specialist clinicians compared with those living in Australia’s metropolitan areas?
	Q9. For individuals with colorectal cancer, are those in non-metropolitan areas less likely to participate in recommended follow-up compared with those living in metropolitan areas in Australia?
	Q10. Are patients with colorectal cancer who reside in non-metropolitan areas more likely to have stomas as part of their treatment than patients residing in metropolitan areas?
Psychosocial outcomes and quality of life	Q11. Do patients who reside in non-metropolitan areas have less support with stomas than patients who reside in metropolitan areas, and does this impact on differences in quality of life?
	Q12. In individuals with colorectal cancer, do those living in non-metropolitan areas have less access to psychosocial care compared to those living in metropolitan areas of Australia?
	Q13. For individuals with colorectal cancer, do those residing in non-metropolitan areas have poorer quality of life after treatment compared with those in metropolitan areas in Australia?
	Q14. For individuals with colorectal cancer, are those who reside in non-metropolitan areas more likely to experience greater psychological distress than those who live in metropolitan areas in Australia?

## Methods

**Fig. 1:**
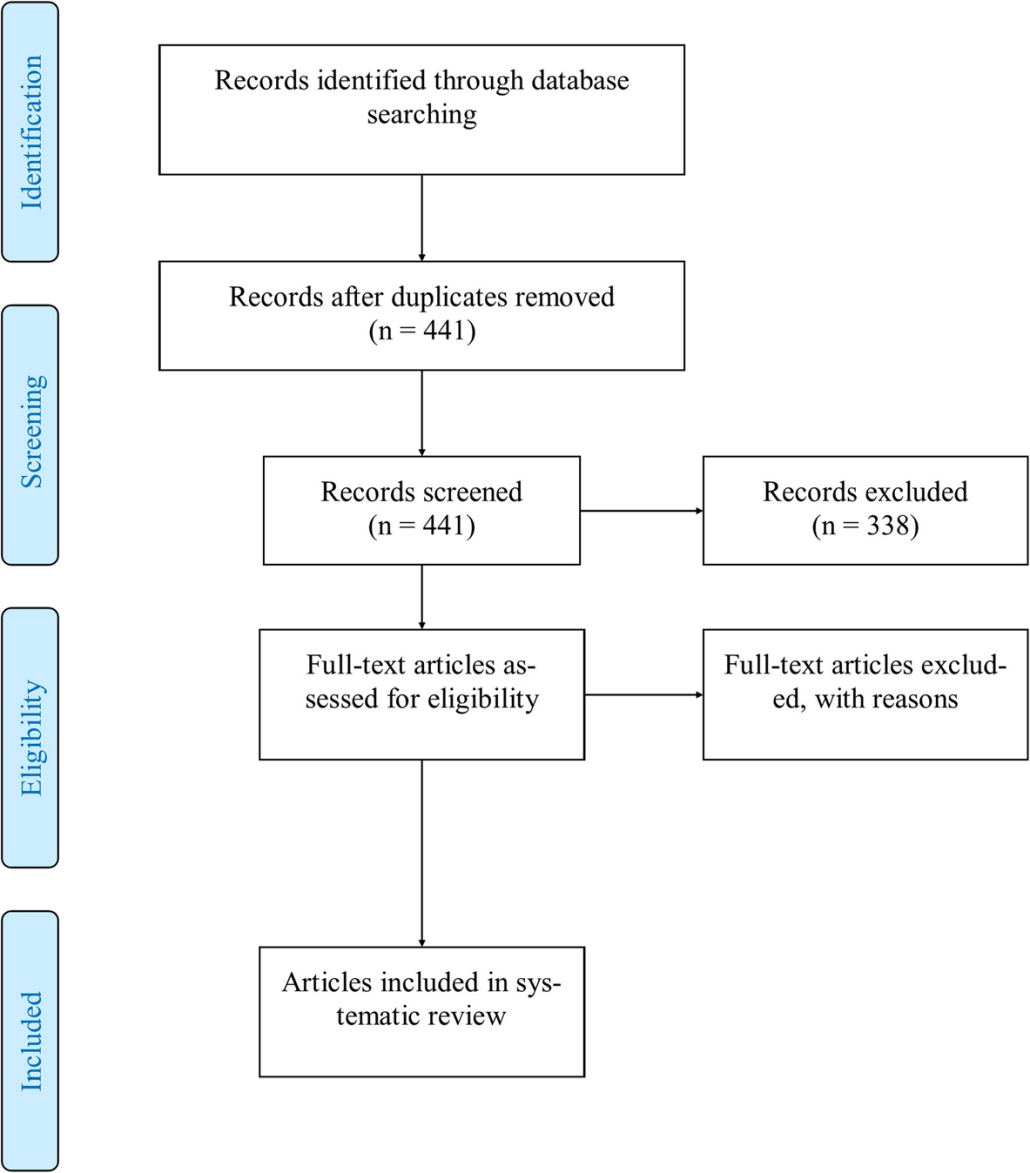
Process of inclusion and exclusion of studies for the systematic review

The review methodology was planned and carried out following the PRISMA statement for the conduct of systematic reviews [19]. The review protocol was registered with PROSPERO; registration number CRD42016042666 (http://www.crd.york.ac.uk/PROSPERO/display_record.asp?ID=CRD42016042666). All stages of the methodology from searching to extraction were carried out by two independent reviewers and discrepancies were moderated by a third independent reviewer. The results of the search and the progression of articles through the screening stages is presented in Fig. 1.

### Search strategy

Searches were conducted in CINAHL, Medline, PsycInfo, PubMed, Embase, ProQuest and Informit. The search covered all articles in these databases between January 1990 and the final search dates of 18^th^ and 20^th^ April 2016. In addition to database searching, manual search methods were also employed to identify potentially relevant articles. This included reference list checks, and identifying key authors or studies in the research area that were known to members of the Project Steering Committee.

Search terms were based on subject headings and key words with separate queries designed for each individual review questions. Search strings all comprised the key terms of “colorectal cancer” or “colorectal neoplasm” or “bowel cancer” or “colon cancer” or “rectal cancer” and “Australia”. Terms relating to geographic disparities included “geographic” or “metropolitan” or “urban” or “rural” or “remote” or “regional”. Additional terms were added for individual clinical questions, such as “survival”, “mortality”, “demographic”, “stage”, “tumour grade”, “screening”, faecal occult”, “clinical management”, “treatment”, “chemotherapy”, “radiotherapy”, “guidelines”, “referral”, “treatment completion”, “follow-up”, “stoma”, “colostomy”, “support”, “psychosocial support”, “quality of life”, and “psychological distress”. Synonyms for these terms were also included in the search strings, and searches were adjusted to best suit the search characteristics of each database.

### Eligibility criteria

Studies were included if they met the following criteria:

1. the data being reported were from Australia;
2. the sample studied individuals with CRC or there was a CRC specific sub-group included in the study;
3. data were reported on outcome measures pertaining to one of the clinical question under review; and
4. data were presented on either:
  - non-metropolitan versus metropolitan comparisons or other geographical inequalities (e.g. low versus high surgical caseload); or
  - a qualitative study on geographical disparities; or
  - a quantitative or qualitative study only for non-metropolitan individuals; or
  - an initiative or intervention designed to address geographical differences in one or more of the outcome measures after CRC diagnosis.

Studies were excluded if they were not available in English, or were review articles, editorials, books, conference abstracts, or commentaries.

### Screening

Screening followed a three-step process: duplicate screening, title/abstract screening, and full-text screening. After removing duplicates, the titles and abstracts were screened for relevance to one of the review questions. In cases where there was insufficient information in the abstract to determine relevance, the article was retained for full-text screening. Articles undergoing full-text screening were checked against the eligibility criteria outlined above.

### Study quality

We utilised an assessment of study quality previously developed for research in breast cancer [20]. This tool was based on an existing valid and reliable tool, the Newcastle-Ottawa Scale (NOS), which assesses the risk of bias in non-randomised observational studies, including case-control and cohort studies [21]; however an alternate scoring system was utilised. Studies were scored according to the extent that they met each of nine criteria using an ordinal scale of 0 (high risk of bias), 1 (intermediate risk of bias), and 2 (low risk of bias). Criteria scores are summed and categorised as “high” (14–18), “moderate” (9–13) or “low” (<9) quality [20].

Qualitative studies were also assessed on nine pre-determined criteria denoting risk of bias [22]. This tool also comprised nine items and articles were graded using the same procedure described above for quantitative articles, with a total possible score out of 18. Mixed methods studies were assessed for methodological quality as qualitative studies as this was the key focus of the study results.

Additionally, we assessed the ‘Level of Evidence for Quantitative Studies’ using published NHMRC criteria [23] where level 1 evidence is considered the most scientifically robust and valid. According to these criteria, a case series, or cross-sectional study is Level IV and a case-control study is Level III-3. A retrospective cohort study is Level III-2 (aetiology) and an unselected or representative case series is Level III-1. A prospective cohort study represents Level II evidence, while Level-I studies are systematic reviews of Level-II studies.

Qualitative studies were likewise assessed on four levels of evidence using published criteria [24]. These levels are: Level I (generalizable studies with conceptual frameworks), Level II (conceptual studies), Level III (descriptive studies) and Level IV (single case studies).

### Geographical classification systems

Several classificatory approaches have been used in the studies reviewed and will be referred to when describing the results of each study. A number of studies adopted the simple distinction of metropolitan versus non-metropolitan, whereby residents of major cities were compared with those residing outside of these cities. More sophisticated approaches have also been adopted that take into account the travelling distance to required treatment centres. Other approaches include the use of a number of formal geographical classifications of which the most common are: the Australian Standard Geographic Classification (ASGC) Remoteness Areas; the Rural, Remote and Metropolitan Areas (RRMA) Classification; and the Accessibility/Remoteness Index of Australia (ARIA).

## Results

From 1990 to 2016, and from an initial pool of 1448 independent articles, 43 published research articles were reviewed. Although seven studies focused on a national Australian population, the majority of studies were state-specific, predominantly from South Australia (11), Queensland (11), New South Wales (9), and Western Australia (4), with one from Victoria. The study characteristics of all 43 studies have been tabulated and published in the Harvard Dataverse open source research data repository [25]. These data can be accessed at http://dx.doi.org/10.7910/DVN/8BTSUP.

Table 2 lists the studies reviewed (organised by the question they provide data on), as well as the study quality ratings. Of the 43 studies included in this review, 38 were quantitative, two were qualitative, and three were mixed methods designs. Data collection methods varied greatly and consisted of population linkage data, survey results, clinical records, focus groups, and interviews. Seventeen of the studies used population-level data from state or national cancer registries.

**Table 2:** Studies included in the review including their quality scores and evidence level

Study	Design	Score	Quality	Level	Question
AIHW (2007) [26]	Quant	15	high	II	1
AIHW (2014) [27]	Quant	14	high	II	1
Baade et al. (2011a) [28]	Quant	16	high	II	1
Chen et al. (2015) [31]	Quant	15	high	II	1
Coory et al. (2013) [32]	Quant	16	high	III-2	1
Cramb et al. (2012) [33]	Quant	17	high	II	1
Roder et al. (2015) [69]	Quant	16	high	II	1
Wilkinson & Cameron (2004) [35]	Quant	9	moderate	II	1
Coory, Ganguly & Thompson (2001) [39]	Quant	15	high	III-2	2
Cramb, Mengersen & Baade (2011) [37]	Quant	13	moderate	IV	2
Homewood, Coory & Dinh (2005) [40]	Quant	15	high	III-2	2
Baade et al. (2011b) [43]	Quant	17	high	III-2	3
AIHW (2015) [12]	Quant	15	high	II	4
Javanparast et al. (2010) [45]	Quant	13	moderate	IV	4
Martini et al. (2011) [47]	Quant	16	high	IV	4
Steffen et al. (2014) [70]	Quant	15	high	II	4
Tong, Del Mar & Kennedy (2000) [71]	Quant	11	moderate	IV	4
Varlow et al. (2014) [48]	Quant	9	moderate	IV	4
Ward et al. (2011) [72]	Quant	16	high	IV	4
Ward, Javanparest & Wilson (2011) [72]	Qual	9	moderate	III	4
Gilbar, Lee & Pokharel (2015) [50]	Quant	11	moderate	III-2	5
Armstrong et al. (2005) [53]	Quant	11	moderate	III-2	6
Armstrong et al. (2007) [54]	Quant	11	moderate	III-2	6
Young et al. (2007) [55]	Quant	14	high	II	6
Morris et al. (2007) [57]	Quant	12	moderate	III-2	7
Goldsbury et al. (2012) [60]	Quant	15	moderate	III-2	8
Emery et al. (2013) [58]	Qual	17	high	III	8
Pascoe et al. (2013) [59]	Qual	15	high	III	8
Ieropoli et al (2011) [63]	Qual	11	moderate	III	12
Dunn et al. (2013a) [15]	Quant	16	high	II	13
Dunn et al. (2013b) [64]	Quant	14	high	II	14
Baade et al. (2013) [29]	Quant	17	high	II	1, 2
Martin et al. (2015) [38]	Quant	11	moderate	III-3	1, 2
Beckmann et al. (2016) [30]	Quant	16	high	II	1, 3
Jong et al. (2004) [34]	Quant	16	high	II	1, 3
Yu et al. (2005) [44]	Quant	16	high	II	1, 3
Hall et al. (2005) [52]	Quant	17	high	II	1, 5
Hocking et al. (2014) [51]	Quant	13	moderate	II	1, 5
Singla et al. (2014) [36]	Quant	14	high	II	1, 5
Wichmann et al. (2013) [62]	Quant	14	high	III-2	1, 9
Armstrong et al. (2004) [42]	Quant	11	moderate	III-2	3, 6, 7
Veitch et al. (2008) [14]	Qual	10	moderate	III	4, 5, 8, 9, 12
Beckmann et al. (2014) [49]	Quant	16	high	III-2	5, 6

Note. *Quant* Quantitative, *Qual* Qualitative

Twelve of the 43 studies were eligible to be included in more than one of the review questions. There were no eligible studies found that addressed question 10 (differences in use of stoma as treatment by residential location), or question 11 (differences in support with stomas by residential location). The following presentation of the results focuses on the broad trends for each question.

### Study quality

The evidence reviewed was generally of high quality. The quality scores and levels of evidence for all included studies are shown in Table 2. Sixteen studies (37%) were of moderate quality, while the majority (*N* = 27, 63%) were high quality, and no studies were low quality. Almost two thirds of quantitative studies were graded as high quality with just over half of these classified as Level II studies, 30% classified as Level III, and 12% classified as Level IV. Two of the five included qualitative and mixed-methods studies were of high quality and all five provided Level III evidence.

### Survival outcomes

For survival outcomes (Q1), 11 of 17 included studies reported significantly poorer survival from CRC for individuals residing outside of metropolitan areas [26–31]. Most of these studies were conducted in South Australia, Queensland, or New South Wales, with limited data available at a national level. Studies that found differences in survival or mortality rates between metropolitan and non-metropolitan areas were all retrospective, population-level studies using cancer registry data. Several studies using ASGC and ARIA methods of geographical classification [26, 27, 31–36] identified poorer survival rates in certain non-metropolitan areas such as ‘inner regional’ or ‘moderately accessible’ areas. Indeed, patients with CRC in ‘remote’ or ‘very remote’ locations often demonstrated better survival than other geographical areas.

### Patient and cancer characteristics

Limited evidence emerged to suggest that patient sociodemographic characteristics (Q2) are implicated in geographical disparities. Of five reviewed studies, one investigated gender [37], two investigated socioeconomic status (SES) [37, 38], and two investigated Indigenous status [39, 40]. There were no studies found that investigated age.

No gender difference was observed in the one study examining CRC incidence between metropolitan and non-metropolitan areas (*p* = 0.693 males; *p* = 0.216 females) [37]. In terms of SES, the role that geographical location plays is still unclear. There was some indication that greater socioeconomic disadvantage was evident amongst non-metropolitan patients with CRC (78%) compared with metropolitan patients (36% disadvantaged) [38], though this is consistent with increased socioeconomic disadvantage in non-metropolitan areas more generally and may be unrelated to CRC [41]. Regarding Indigenous status, two studies [39, 40] included in this review reported a lower incidence of CRC in Indigenous populations in discrete rural and remote areas, compared to the national average (age-standardised incidence rates of 20–40).

This review found a lack of evidence (only one of five reviewed studies) to support differences in stage at diagnosis between patients with CRC from metropolitan and non-metropolitan areas (Q3). Three of the five reviewed studies found significant variations in stage at diagnosis when examining colon and rectal cancer independently, with colon cancer often diagnosed at a later stage than rectal cancer, though this was irrespective of geographical location [42–44].

### Diagnostic and treatment characteristics

#### Screening participation

Studies that focused on screening participation (Q4) generally suggest that greater numbers of women and more affluent individuals participated in CRC screening, regardless of geographical location. Furthermore, significantly lower screening participation was observed in areas known to have large Indigenous populations and populations with and low socioeconomic status [45]. However, no significant differences emerged in comparisons of metropolitan and non-metropolitan areas generally or in barriers to screening. Five of the nine studies investigated screening participation across a range of geographic areas rather than collapsing into metropolitan and non-metropolitan areas, with two of these studies reporting higher rates of participation in inner regional or rural residents (eg. Martini *et al:* 48.6%), whereas individuals in remote locations had equivalent participation rates (46.0%) to metropolitan residents (45.6%) [46, 47].

#### Knowledge about screening

Important geographical differences in knowledge about bowel cancer screening were evident in one study [48]. The results showed a higher proportion of individuals in metropolitan areas believing that screening is only necessary when experiencing symptoms (23.3% vs 16.6% non-metropolitan).

#### Clinical management

Regarding clinical management (Q5 & 6), three studies were identified that investigated geographic disparity in chemotherapy [49–51], three studies focused on surgery [36, 51, 52], one study focused on access to treatments [14], and five studies investigated adherence to treatment guidelines [42, 49, 53–55]. One noteworthy omission was the lack of evidence with regards to radiotherapy use in non-metropolitan areas. Only two studies reported geographical differences for this question. For example, Beckman and colleagues [49] reported that chemotherapy was less likely to be received by rural patients with stage III colon cancer (Prevalence Ratio = 0.87), and Hocking and colleagues [51] found increased use of combination chemotherapy in metropolitan patients (67.4% v 59.9%; *p* = 0.01).

#### Deviation from recommended clinical management

From five available studies, there was limited evidence to conclude that deviation from recommended clinical management occurred as a function of geographic location (Q6). Generally, overall treatment received was consistent with national guidelines and similar across geographic locations, although one study reported overall discordance with clinical guidelines was more likely for patients residing in rural areas (prevalence ratio 1.2) [42]. There was some evidence to suggest that rates of chemotherapy in patients with stage III CRC tended to be lower in remote areas, as did preoperative examinations [42, 49]. However, there was generally insufficient research available to address this question, and some of this evidence (the NSW Colorectal Cancer Care Survey; [42, 53, 54] relied on practitioner-self-report rather than archival records. Further difficulties in determining geographic disparities emerged as a result of most studies not reporting whether rural and remote patients were receiving treatment in regional centres or metropolitan centres, nor did they examine the effect of this on outcomes. This may be particularly important given findings for Question 1 of more favourable outcomes for patients from remote and very remote regions who are required to travel for treatment.

#### Completion of treatment

Only two studies were found that investigated geographical differences in the completion of prescribed treatment (Q7). Remote patients were less likely to complete radiotherapy yet more likely to complete chemotherapy treatment than patients in other areas [42]. This may be due to access to radiotherapy facilities [56]. Treatment completion was also shown to be poorer for patients with greater area-based disadvantage (52.6% vs 76.1%), measured using the Socio-Economic Indexes for Areas (SEIFA; [57]). These studies did not report reasons for these discrepancies.

#### Referral

There were only four studies that investigated geographical variation in referral delays (Q8), three of which did not directly compare geographical locations [14, 58–60]. Diagnostic delays in cancer were found to generally become more common with increasing rurality, due to an undersupply of medical practitioners in these areas [61]. The reviewed studies highlight potential inadequacies in the referral process for CRC patients in non-metropolitan areas. However, none provided a direct comparison of referral times between CRC patients from different regions. Alternative factors such as private health insurance status and GP-specialist relationships were shown to impact on referral times [59].

#### Follow-up

The sparse evidence (two studies) on participation in recommended follow-up (Q9) suggests a willingness from non-metropolitan patients to comply with recommended follow-up and suggests that rates of follow-up for these patients is high [14, 62]. Generally there is insufficient data to draw definitive conclusions for this question.

#### Use of stomas in treatment

The current review found no articles addressing question 10 regarding geographical differences in patients’ receiving stomas as part of treatment.

### Psychosocial outcomes and quality of life

There were few studies focusing on psychosocial outcomes and support of patients with CRC. For example, no studies were found that addressed question 11 (geographical variation in support with stomas and the impact on quality of life) of this review.

Reviewed studies provide limited evidence to determine whether there are geographical disparities in psychological and social support, quality of life, and psychological distress in patients with CRC. Only two mixed-methods studies were found that investigated psychosocial support received by non-metropolitan patients with CRC (Q12). These studies suggest that rural patients typically looked to GPs and peers to meet their support needs [14, 63]. There was, however, no direct comparison to the support received by metropolitan patients. From the available evidence, we are unable to determine the level of access or use of psychosocial care services for patients with CRC generally, or across different geographical regions.

Only a single study was located examining geographic differences in quality of life for CRC patients (Q13). This study found that remoteness of residence predicted poorer outcomes in a cancer specific quality of life domain (OR = 0.42; no differences emerged on physical, functional, social/family, and emotional well-being domains) [15].

Only one study was located which examined patterns of psychological distress in CRC survivors (Q14). This study found that psychological distress at various time points post treatment did not differ by geographic location [64].

## Discussion

This review found consistent evidence to suggest that issues such as screening and early detection, socio-demographic characteristics, tumour characteristics, treatment options, and access to oncology services play a complex role in shaping geographical disparities in survival. The majority of studies examining survival were high quality, providing level II evidence. However, studies that were of only moderate quality were more likely to report no significant difference in survival between metropolitan and non-metropolitan CRC patients.

Some of the factors under review appeared to interact with each other. For example, while SES is recognised as a risk factor for reduced cancer survival, research suggests that this may be due to treatment and healthcare system factors (e.g. private versus public), as survival for patients with CRC given equivalent treatment does not appear to depend on SES [65, 66]. The reviewed evidence also suggests that deviations from clinical management guidelines may be more likely to be influenced by patient (age, gender, Indigenous status, and health insurance status) and system-level factors (access, centre waitlists, and surgeon or hospital case-loading) than by geographical location. Furthermore, regarding adherence to prescribed treatment, patients may themselves choose approaches that minimise disruptions to their lives (such as moving to a larger town during treatment) and this is likely to vary across patient and cancer characteristics.

Evidence for disparities in survival uncovered by this review is consistent with trends for other types of cancer in Australia [27]. Although somewhat inconsistent, the evidence generally supports the assertion that survival is poorer for patients with CRC in areas outside of major cities, and is likely moderated by a range of contributing factors.

**Fig. 2:**
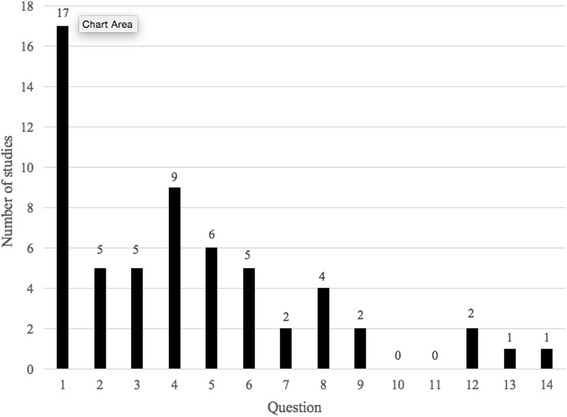
Distribution of studies for each clinical question

We anticipated that the reviewed evidence would highlight patterns of covarying disparities across different factors and outcomes, and that this would shed light on possible causes of these disparities. Unfortunately there was insufficient evidence to achieve this. Across this body of research, despite evidence of some geographical disparities, there was generally a lack of clear, consistent findings on the nature of these disparities and how they manifest for patients with CRC. The evidence reviewed was inconsistent, or in some cases completely lacking. The distribution of studies identified through our search is displayed in Fig. 2. Geographical disparities in psychosocial support, quality of life, and psychological distress are not adequately addressed in the published literature to date. Likewise, research into geographical variations in adherence to prescribed treatment and recommended follow-up is also limited. Additionally, evidence for geographical disparities in the use of stomas as part of treatment, and the provision of support with stomas, is non-existent.

The majority of published research has focused on Question 1 and Question 4. Survival and screening have to date been the topics of greatest interest to researchers investigating the Australian context. These topics might also have garnered the most attention due to the fact that major data registries exist that store information relating to survival and screening. Given difficulties in collecting sufficient samples to draw meaningful comparisons across populations, it is no surprise that researchers have tended towards addressing research questions for which data already exists.

The challenge remains to identify data sources for the under-researched issues identified by this review. There may be opportunities to augment existing data collection efforts that are underway to support the large state and territory registries. For example, a short distress instrument like the Distress Thermometer or K6 [67, 68] could be routinely administered at the time information is being recorded for the Australian state and territory cancer registries. This would allow an examination of the magnitude of psychological distress experienced by individuals with different individual characteristics and across geographical location of residence/treatment.

Despite the largest number of studies focusing on survival disparities, 35.3% (6/17) of these have grouped non-metropolitan regions together for comparison with metropolitan centres, and thus, should be considered low in resolution. Evidence suggests this approach may lack sensitivity for uncovering meaningful disparities between non-metropolitan regions. If, as the current evidence highlights, it is inner regional areas that are at the greatest disadvantage and that rural and remote areas appear more similar to metropolitan centres (at least in survival), then understanding these differences could potentially shed light on currently unknown causes of disparity. Furthermore, researchers run the risk of concluding that no geographical disparity exists if all non-metropolitan regions are collapsed together since this will conceal important differences between metropolitan centres and specific region-types. The state and territory cancer registries record statistical local area and patient postcode at diagnosis and therefore, we recommend this data be used for more fine-grained analysis.

We recognise that the formulation of our review questions also targeted broad metropolitan versus non-metropolitan discrepancies, though our analysis was able to distinguish between region-types in cases where primary data at this level was reported. We recommend future studies take a fine-grained approach to classifying and analysing regional differences, or at minimum collect detailed information regarding precise location of residence and treatment received so that future analyses are able to capture disparities more accurately. We expect that in coming years, more focused examination of patient-, professional-, and system-level factors will allow for the development of a more comprehensive framework or instrument that can explain disparities based on specific locations (e.g. postcode) rather than regions (e.g. inner regional).

### Limitations

The current review was limited by several factors. The most notable being the absence of sufficient evidence to address a number of the review questions. Our ability to synthesise the available evidence was also limited by the variation in design, methodology, samples, analysis, and presentation of results of the included studies. However, given the limited number of studies available in the area, and the aim of this review to provide an overview of the literature within each of the themes, it was important to include all available evidence regardless of design. Additionally, there was significant variation in the use of geographic classification systems, which was further complicated as some studies combined all non-metropolitan locations into a single category while others analysed up to five categories of remoteness.

Variations in study quality also prohibited clear conclusions. Factors that reduced quality scores in quantitative studies were: not using a representative sample, not adjusting for potential key confounders, inadequate or unclear handling of missing data, and poor follow-up. For qualitative studies, factors that reduced quality included: not addressing interviewer bias, unclear data recording methods, no rationale for sample size, and inadequate description of sample.

Finally, the methodology of the review itself was limited in only reviewing published research. There may be grey literature, theses, conference proceedings, or industry reports that report data bearing on the questions posed by this review.

## Conclusions

This review found that patients with CRC in regional, rural, and remote areas of Australia have a poorer survival rate and experience less optimal clinical management; however, this evidence is limited and at times inconsistent. Further, access to treatment and services was not always the main driver of disparities, with individual patient characteristics and type of region also playing an important role. There is an urgent need for more research to be conducted, particularly with respect to rates of treatment completion, adherence to recommended follow-up, experience of stomas, psychosocial care, psychological distress and quality of life. This review also highlights the need for geographical disparities in the care of patients with CRC to be more thoroughly analysed as the interrelationships between distance to services, demographic factors, and patient outcomes are evidently complex. The challenge for governments and health service providers is to find a way in which best practice in prevention, early diagnosis and ongoing management of CRC and associated psychosocial needs can be made available in all non-metropolitan areas, and targeted towards the specific needs of different metropolitan and regional populations.

## Abbreviations

ARIA: Accessibility/Remoteness Index of Australia
ASGC: Australian Standard Geographic Classification Remoteness Areas
CRC: Colorectal cancer
FOBT: Faecal Occult Blood Test
GP: General practitioner
NBCSP: National Bowel Cancer Screening Program
NOS: Newcastle-Ottawa Scale
RRMA: Rural, Remote and Metropolitan Areas Classification
SES: Socioeconomic status.
